# P-1921. Risk Factors for Hospitalization due to COVID-19 in the Current Era of the Pandemic

**DOI:** 10.1093/ofid/ofae631.2081

**Published:** 2025-01-29

**Authors:** Bhavya Ancha, Keith W Hamilton, Lauren Dutcher, Kathleen Degnan, Kurt Palumbo, Amanda Binkley, Sahrish Ilyas

**Affiliations:** University of Pennsylvania, Phiadelphia, Pennsylvania; University of Pennsylvania Perelman School of Medicine, Philadelphia, Pennsylvania; University of Pennsylvania Perelman School of Medicine, Philadelphia, Pennsylvania; University of Pennsylvania Perelman School of Medicine, Philadelphia, Pennsylvania; University of Pennsylvania, Phiadelphia, Pennsylvania; Penn Presbyterian Medical Center, North Wales, Pennsylvania; University of Pennsylvania, Phiadelphia, Pennsylvania

## Abstract

**Background:**

Certain risk factors place SARS-COV-2 infected individuals at a higher risk of hospitalization and death. While increased vaccination rates and herd immunity have decreased the overall risk of hospitalization and death from COVID-19, some groups of patients may still be at significant risk of morbidity and mortality from COVID-19. However, this population has not been well-described. This study sought to understand the current landscape of patients hospitalized due to COVID-19 to gauge who would most benefit from pre-exposure prophylaxis and other therapies for COVID-19.

**Table 1:**
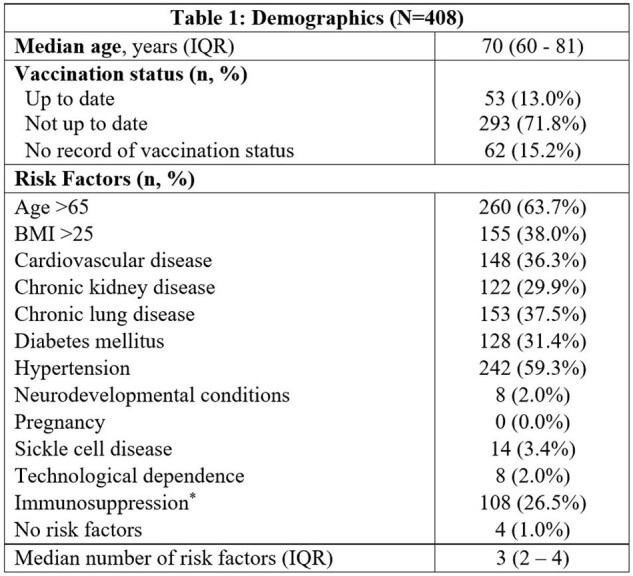
Demographics. Demographics of patients admitted to hospital due to COVID-19. *Immunosuppression defined as having at least one of the following: hematologic malignancy, solid organ transplant, bone marrow transplant, metastatic cancer, asplenia, HIV with CD4 count < 200, other immunosuppressive disease with impaired humoral or cell-mediated immunity, or immunosuppressive medication including corticosteroids (prednisone ≥ 2mg/kg/day or ≥ 20 mg/day or equivalent dose of other corticosteroid) for > 14 days, chemotherapy within past 3 months, calcineurin inhibitor, anti-proliferative agent, mTOR inhibitor, B-cell or T-cell depleting medication, or TNF-alpha inhibitor.

**Methods:**

This was a retrospective study of adults admitted to a single center for a COVID-19-related reason from August 4, 2023, through February 5, 2024. All admitted patients with a positive SARS-CoV-2 test were identified; patients who were admitted for a non-COVID-19 related reason were excluded from manual chart review. Patient demographic information, vaccination status, risk factors, symptoms, hospital course, and treatment interventions were collected. These data were then analyzed and descriptively characterized.

**Table 2:**
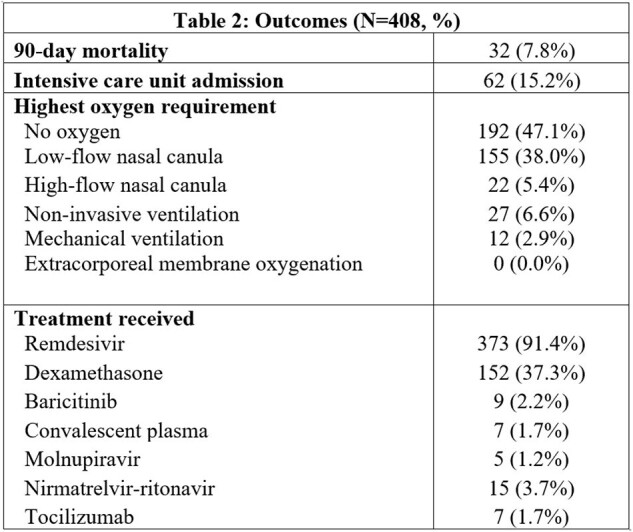
Outcomes Clinical outcomes of patients admitted to hospital due to COVID-19.

**Results:**

Of the 408 patients identified who were hospitalized for a COVID-19-related reason during the study period, the median age was 70 years (IQR 60 – 81) with a median length of stay of 5 days (IQR 3-8). Only 13% of patients were up to date on their COVID-19 vaccination at their time of admission (Table 1). Admitted patients had a median of 3 (IQR 2 – 4) risk factors for severe infection, and 26.5% of patients were noted to be immunosuppressed (Table 1). Nearly half of all patients did not require supplemental oxygen during their stay (47%); 38% required only low flow nasal cannula (Table 2). There was a 7.8% 90-day mortality rate.

**Conclusion:**

These data suggest that most admitted patients in the current COVID-19 era have multiple risk factors and are under-vaccinated. This high-risk population may benefit from updated vaccination, pre-exposure prophylaxis, and/or other therapies to reduce the morbidity from COVID-19.

**Disclosures:**

Kathleen Degnan, MD, Gilead: Grant/Research Support Amanda Binkley, PharmD, BCIDP, AAHIVP, Viiv: Advisor/Consultant

